# Appalachian Environmental Health Literacy: Building Knowledge and Skills to Protect Health

**DOI:** 10.13023/jah.0201.06

**Published:** 2020-01-26

**Authors:** Anna G. Hoover, Annie Koempel, W. Jay Christian, Kimberly I. Tumlin, Kelly G. Pennell, Steven Evans, Malissa McAlister, Lindell E. Ormsbee, Dawn Brewer

**Affiliations:** University of Kentucky College of Public Health, anna.hoover@uky.edu; University of Kentucky Department of Dietetics and Human Nutrition, ann.koempel@uky.edu; University of Kentucky College of Public Health, jay.christian@uky.edu; University of Kentucky College of Public Health, kimberly.tumlin@uky.edu; University of Kentucky College of Engineering, kellypennell@uky.edu

**Keywords:** environmental health, environmental health literacy, risk communication, health communication, Appalachia

## Abstract

Environmental health literacy (EHL) is an emerging, multidisciplinary field that promotes understanding of how environmental exposures can affect human health. After discussing the regional relevance of environmental health knowledge and skills, this article describes three ongoing Appalachian projects that are focused on measuring and building EHL.

## INTRODUCTION

Well-documented health disparities in Appalachia include high incidence of diabetes, obesity, high blood pressure, and cancer.^1^–^3^ The most recent release of *America’s Health Rankings* places Central Appalachia in particular near the bottom of all U.S. states in health outcomes, with Tennessee 42nd, West Virginia 44th, and Kentucky 45th among the 50 states.^4^ Similarly, *County Health Rankings* places six of Virginia’s seven Central Appalachian counties in the state’s bottom quartile for health outcomes.^5^

Many chronic health conditions disproportionately experienced across the region have been linked to environmental contaminants.^6^–^8^ Central Appalachia’s historical economic reliance on mining, agriculture, and other industries has provoked concerns about how environmental exposures may contribute to regional health disparities. Research has found heightened levels of sulfur dioxide and other acidic particles in air samples,^9^,^10^ while community members have voiced water quality concerns in several studies.^11^–^13^

Deteriorating infrastructure,^14^ inappropriate waste disposal,^15^ and potential occupational exposure risks^16^ compound the need for at-risk populations to receive clear, timely, and accessible information about potential environmental health threats. Transforming complex scientific evidence into useful, understandable, and readily available resources and tools is essential to helping the people and communities of Appalachia make informed, health-protective decisions about their environment.

Environmental health literacy (EHL) is an emerging field that brings together content and methods from health, social, and environmental sciences to promote understanding of how environmental exposures can affect human health.^17^,^18^ Such understanding can spur actions to minimize exposures and improve health outcomes. Local health department staff, healthcare providers, Cooperative Extension agents, librarians, journalists, and others play critical roles in sharing evidence-based information that can help build EHL.

Land-grant institutions are well positioned to help Appalachian organizations access and share information to improve regional EHL. The University of Kentucky (UK), for example, houses many initiatives that address regional environmental, health, and socioeconomic challenges. Spearheaded by faculty and staff from the Colleges of Public Health and Engineering, the Department of Dietetics and Human Nutrition, and the Kentucky Water Resources Research Institute, UK teams are conducting three NIH-funded, stakeholder-engaged studies that strive to measure and build regional EHL.

**Assessing EHL in Appalachia** is a pilot study supported by the National Institute of Environmental Health Sciences (NIEHS) through the UK Center for Appalachian Research in the Environmental Sciences (UK-CARES) that addresses the intersection of EHL and water quality. Researchers are leveraging local knowledge from water utility operators and watershed volunteers to identify the knowledge and skills people need to protect their health in the event of water contamination. This study will produce one of the first validated EHL measurement instruments.

**Protect Your Body from Pollution with a Healthy Lifestyle (Body Balance)**, a project of the NIEHS-funded UK Superfund Research Program, seeks to increase EHL by educating people about food strategies that may reduce exposures and decrease harmful exposure-related health effects.^19^ Implemented by Cooperative Extension Service Family and Consumer Sciences (FCS) agents, the seven-lesson curriculum encourages consumption of plant-based foods believed to decrease inflammation and oxidation linked to exposures.^20^ The curriculum also encourages consumption of lean meats and dairy products as high-fat animal products tend to contain higher concentrations of pollutants.^21^ Body Balance is available to FCS agents in all 120 Kentucky counties.

**Integrating Information Resources to Increase EHL in Appalachian Eastern Kentucky**, funded by the National Library of Medicine, finds investigators working with Eastern Kentucky residents—including public health and healthcare stakeholders—to assess and improve existing environmental health-related risk maps and other information resources. The study answers calls to bridge knowledge and skills gaps by bringing together scientists, regulatory agencies, and community members to develop and disseminate accessible, understandable, and useful information.

At its roots, EHL is context-specific. Communities face diverse health risks from assorted contaminants traveling varied pathways to expose different populations, and these risks must be addressed within unique social, economic, and political contexts. Community residents understand these contexts far better than anyone else. By taking a stakeholder-engaged approach to EHL, the projects described in this article are harnessing local knowledge to help ensure that people in the Appalachian region have the evidence-based, culturally competent guidance that they need to make decisions about both their health and their local environment.

**SUMMARY BOX**

**What is already known about this topic?** Appalachia’s history of health disparities, along with potential linkages between some diseases and environmental contaminants, underscores the need for residents to have information, knowledge, and skills to protect themselves from potential environmental health threats. Local health departments and other community organizations play key roles in sharing information about these topics.

**What is added by this report?** This report describes three ongoing projects designed to build environmental health literacy and improve available environmental health information resources in the region.

**What are the implications for public health practice, policy, and research?**

Stakeholder-engaged approaches can increase access to better information and help build environmental health literacy in Appalachia, ultimately leading to evidence-informed individual and community decisions.
